# An Integrated Multi-Disciplinary Perspective for Addressing Challenges of the Human Gut Microbiome

**DOI:** 10.3390/metabo10030094

**Published:** 2020-03-06

**Authors:** Rohan M. Shah, Elizabeth J. McKenzie, Magda T. Rosin, Snehal R. Jadhav, Shakuntla V. Gondalia, Douglas Rosendale, David J. Beale

**Affiliations:** 1 Department of Chemistry and Biotechnology, Faculty of Science, Engineering and Technology, Swinburne University of Technology, Hawthorn, VIC 3122, Australia; rshah@swin.edu.au; 2 Land and Water, Commonwealth Scientific and Industrial Research Organization (CSIRO), Dutton Park, QLD 4102, Australia; 3 Liggins Institute, The University of Auckland, Grafton, Auckland 1142, New Zealand; liz.mckenzie@auckland.ac.nz (E.J.M.); m.rosin@auckland.ac.nz (M.T.R.); 4 Centre for Advanced Sensory Science, School of Exercise and Nutrition Sciences, Deakin University, Burwood, VIC 3125, Australia; snehal.jadhav@deakin.edu.au; 5 Centre for Human Psychopharmacology, Swinburne University of Technology, Hawthorn, VIC 3122, Australia; sgondalia@swin.edu.au; 6 Anagenix Ltd., Parnell, Auckland 1052, New Zealand; doug.rosendale@anagenix.com

**Keywords:** metabolomics, microbiome, omics integration

## Abstract

Our understanding of the human gut microbiome has grown exponentially. Advances in genome sequencing technologies and metagenomics analysis have enabled researchers to study microbial communities and their potential function within the context of a range of human gut related diseases and disorders. However, up until recently, much of this research has focused on characterizing the gut microbiological community structure and understanding its potential through system wide (meta) genomic and transcriptomic-based studies. Thus far, the functional output of these microbiomes, in terms of protein and metabolite expression, and within the broader context of host-gut microbiome interactions, has been limited. Furthermore, these studies highlight our need to address the issues of individual variation, and of samples as proxies. Here we provide a perspective review of the recent literature that focuses on the challenges of exploring the human gut microbiome, with a strong focus on an integrated perspective applied to these themes. In doing so, we contextualize the experimental and technical challenges of undertaking such studies and provide a framework for capitalizing on the breadth of insight such approaches afford. An integrated perspective of the human gut microbiome and the linkages to human health will pave the way forward for delivering against the objectives of precision medicine, which is targeted to specific individuals and addresses the issues and mechanisms in situ.

## 1. Introduction

Recent advances in culture-independent study techniques of microbial communities, as well as an increasing interest in the role of the gut microbiota in health and disease, have facilitated vast insights into human microbial communities [1]. Identification of prokaryotes mainly with 16S ribosomal RNA (rRNA)-encoding gene sequences [2] and eukaryotes with predominantly Internal Transcribed Spacer (ITS) rDNA sequences [3], coupled with metagenomic analyses, has revealed the ever-increasing diversity list of microorganisms within the human gut [4]. Transcriptomic, proteomic and metabolomic high-throughput tools allow us to begin to grasp the function of the human gut microbiome [5]. Despite these tools, the current state of microbiome research struggles to account for all members of the microbial community, integrate community structure with function, and characteristics of spatial and temporal aspects of the gut microbiota ecosystem [4,5,6]. Navigating these challenges further complicates our primary task: to design and carry out experiments that generate data which can be integrated, analysed, and interpreted to yield biologically relevant findings for human health.

Most studies tend to focus exclusively on the bacterial component of the human gut, likely omitting the archaea, eukaryotes (including fungi) and viruses within the gastrointestinal tract [7], which has a potential to miss significant microbial intra- and inter-kingdom interaction, and calls into question the relevance of diversity scoring, which is widely reported and used as a consolidated and simplified metric for a vast and complex ecosystem. In the interest of understanding function and mechanisms, microbial metabolites have become a target of investigations, and a sincere international effort has been made to create databases that allow function to be inferred with relatively high confidence from a range of types of sequence data. Inferential tools are invaluable in interpreting high-throughput cost-effective taxonomical datasets, but the gold standard for determining function remains chemometric detection of small molecules in biological samples, which presents additional complications to be accounted for.

Most samples used in the study of the human gut microbiome are home-collected after defecation by the participant rather than endoscopically-collected from the gut since the cost and feasibility of self-collection outweighs in vivo procedure [8]. Faecal samples provide data on the distal microbiome of the gastrointestinal tract (GIT), which is not representative of the proximal microbiome. Although metagenomics of home- and endoscopically-collected samples show little variation between the samples, volatile organic compounds (VOC) in the metabolome differ between the in vivo and ex vivo faecal samples [8], which is important when considering the self-collected faecal samples metabolome as a proxy for the functional reflection of the gut microbiome [9].

Despite these fundamental limitations, either metabolic activity initiated by the host or the gut microbiota can lead to marker metabolites in different biological fluids that allow differentiation between healthy and disease status. Metabolomics may be viewed as a process in which non-targeted metabolomics, or global metabolite profiling, aims to discover a list of candidate metabolites of interest, or biomarkers, which can be further quantified and validated using targeted metabolomics. Accounting for vast individual variation in the microbiome and metabolome has led to the development of alternative experimental designs, such as ‘N of 1,’ which bypasses the challenge of increasing noise while attempting to increase a signal by increasing the ‘N’ of a study. Similarly, this approach addresses the challenge of applying traditional statistics based on power calculations and reproducibility to the multivariate and non-parametric datasets that represent microbiome structure and function. At present, most microbiome findings present correlations rather than causation, which can only be overcome with a solid grasp on the functional relationship between host and microbiome.

To further investigate different perspectives of this area, a peer session on ‘metabolomics and its application in gut microbiome research’ was held during the most recent Australian and New Zealand Metabolomics (ANZMET) conference (Auckland, New Zealand, from 30 August to 1 September 2018). Over 20 metabolomics-based researchers attended the session and participated in the discussion on how functional omics could be used to advance gut microbiome research. In addition to summarizing the key points from the peer session, we provide an overview of the current challenges when researching the gut microbiome, from the perspective of the metabolomics community. In should be noted that challenges relating to specific sampling and storage conditions or techniques and approaches are not captured in this review. Where possible, the interested reader is directed to other published works for this information.

## 2. Biogeography of the Microbiome and Metabolome: Implications for Faecal Samples as Proxies

Human cells live in coexistence with a vast and diverse collection of symbiont microorganisms referred to as the human microbiota or the microbiome. The GIT is, by far, the most heavily colonized organ in the human body, with a large surface area and a consistent nutrient source of digested food for microbes to utilise, making it a preferred site for microbial colonisation. The GIT is also one of the most studied microbial ecosystems [10,11,12,13]. Although research to date has mainly focused on the bacterial component of the human gut, microorganisms belonging to Archaea and Eukarya domains of life, as well as viruses, also constitute the human microbiome [14,15,16]. A rich and complex ecosystem of bacteria, fungi, viruses, archaea, protists and (sometimes) helminths flourishes in the human gut.

**Figure 1:**
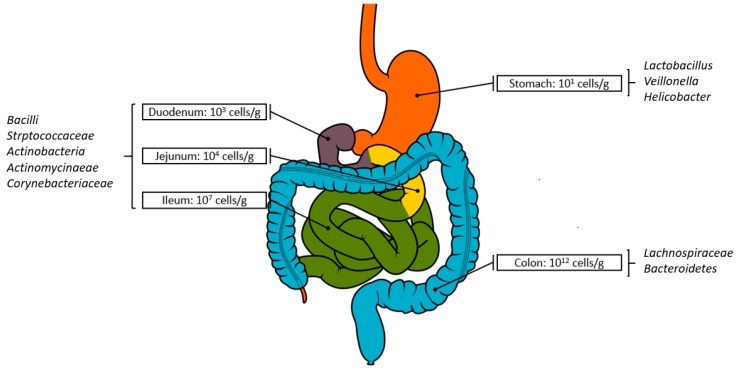
Variations in bacterial number and composition across the length of the gastrointestinal tract (GIT). Image: Olek Remesz (wiki-pl: Orem, commons: Orem) (https://commons.wikimedia.org/wiki/File:GISystem.svg), “GISystem“, Text modified/overlaid by Shah et al., https://creativecommons.org/licenses/by-sa/2.5/legalcode.

Among the trillions of microorganisms that make up the gut microbiome, commensal bacteria are predominant and distributed throughout the GIT. Recent endeavours in gut microbiome research using metagenomics has provided a strong understanding of bacterial communities in largely diverse environments [17,18,19,20,21,22,23]. Although composed of strict anaerobic bacteria from over 50 different phyla, the *Firmicutes* and the *Bacteroidetes* are the two dominant phyla in the human gut. The members of other phyla such as *Proteobacteria*, *Verrucomicrobia*, *Actinobacteria*, *Fusobacteria*, and *Cyanobacteria* are present in minor proportions. The number of bacteria increases from 10 cells/g of contents in the stomach to 10^12^ cells/g in the colon (Figure 1). The human gut microbiota is not homogenous and as such, a wide variation in microbial composition between these sites is reported (Figure 1).

The degree of richness, complexity and function of the regional gut microbiome is highly dependent on the microenvironmental conditions, nutrients, oxygen and water availability, as well as host site-specific characteristics [24]. The lowest microbial counts are observed in the stomach and upper small intestines, due to localized harsh conditions (i.e., high acidity, high bile acid concentrations and short retention time) [25]. A gradual increase in microbial numbers towards the distal ileum and within the colon reflects a more tolerant microenvironment that permits colonization [26]. Bacteria residing in the human colon are thought to be the most substantial contributors to the total human microbiome population, with an estimate of 3.8 × 10^13^ cells [27] that influence physiological processes, both in health and disease [2].

Faecal samples are used as proxies for the fermenta within the colon. Highly developed biofilm microbiota closely associated with the intestinal mucosa are generally believed to be more relevant than the planktonic microbes that exist in the lumen gut. As such, faecal samples may not present an accurate snapshot of the mucosal microbiota. This is a necessary compromise in the absence of routinely used endoscopy to sample the contents of the colon, even though we know that endoscopically collected (in vivo) samples have different microbiome profiles and markedly different metabolome profiles than faecal (ex vivo) samples [8]. Endoscopic biopsies also have their own limitations, such as fasting and colon cleansing prior to endoscopic collection, and the contamination of tissue specimens by luminal microbes during the process. Faecal sampling is non-invasive, and thereby more accessible to researchers. The treatment and handling of samples after collection is a critical aspect of ongoing microbiome studies. Lauber, et al. [28] studied the effects of storage conditions on human faeces using 16s rRNA pyrosequencing. The results indicated that the microbial composition was not significantly affected by short-term storage of up to 14 days at −80 °C, −20 °C, 4 °C and 20 °C. In another study, Wu, et al. [29] showed that there was no significant difference between the faecal samples immediately frozen at −80 °C and those stored on ice for up to 48 h. On the contrary, Roesch, et al. [30] revealed that the stability of faecal samples may be compromised when stored at room temperature for more than 12 h. For more information on the collection and storage of microbiome related samples, the reader is directed to the reports by Panek et al. [31] and Vanderputte et al. [32].

**Figure 2:**
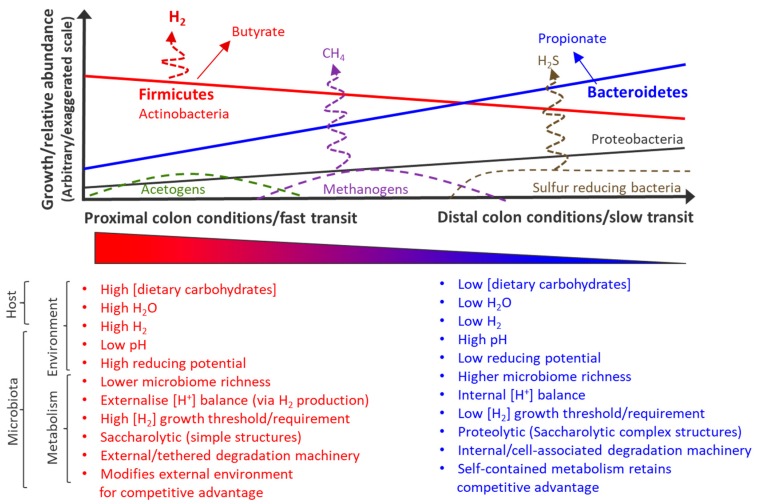
Schematic representation of conditions gradient in the colon determining state of gut ecosystem and how this effects faecal snapshots. Local/regional conditions in the gut graphically represented, left to right, from entry (proximal colon) to exit (distal colon).

However, despite their availability, we need to consider how and what faecal samples proxy for. Faecal samples are not end-points of a determinative process, they are snapshots of a continuously developing and evolving microbial ecosystem, where the ecological stage encapsulated in that snapshot faecal sample is driven by transit time and stool consistency (water content) [33] Differences in transit represents different rates of passage of material through the colon, and hence different ages of the developing ecology, and these ages in turn are representative of the physical, chemical and microbiological continuum throughout the length of the colon. We summarize these conditions in Figure 2, where faecal snapshots could vertically bisect any given point on the continuum. Thus, faecal samples may proxy for quite different gut conditions.

The implications of this are that different relative abundances of microbes and their respective metabolites are present. Firstly, this may explain much of the inter-individual differences observed in microbiome research, and furthermore, simply explain much intra-individual differences over time. As an aside, the intersubject variation in microbial metabolites argues in favour of cross over designs in intervention studies and, therefore, individuals can act as their own controls. Nevertheless, if these snapshot dynamics can be modelled and standardized, we would be able to account for much of the observed variation (at least at a metabolic or ecological niche or functional microbial guild level). Potentially, we may unmask common mechanisms thus far obscured by variation. Secondly, this means that with different relative abundances of microbes, metabolite profiles will obviously be different. The most obvious consequence of this at the bacterial phylum level is that a snapshot representative of more proximal colonic conditions rich in Firmicutes would be higher in butyrate and lower in propionate, whilst a snapshot representative of more distal conditions proportionately richer in Bacteroidetes would have higher concentrations of propionate and likely be comparatively depleted in butyrate (Figure 2). Obviously, other metabolites would also be different, as befits the other interconnecting microbial- and host-driven environmental conditions (Figure 2). This is commonly observed with the other fermentation by-products.

To illustrate this, as a part of core metabolism, fermentation of sugars through glycolysis has the cofactor NAD^+^ converted to NADH + H^+^. Regeneration of NAD^+^ can become the rate-limiting step in glycolysis, so is balanced by the rapid regeneration and commensurate generation of lactate, acetate, formate, succinate and ethanol [34]. Thus, acid production is a necessity driven by competition for abundant energy resources. Bacteria in a dietary carbohydrate-richer environment will maintain their competitive advantage by rapidly pushing sugar-derived carbons through glycolysis and out as waste products to deny their competitors that same raw resource. Alternative pathways will be employed as variations of this theme, where trade-offs between energy yield and protein cost are made [35]. In vitro experiments show that under the right carbohydrate-rich conditions, a single bacterium can dominate a faecal microbiome, with commensurate impact on the short-chain fatty acid (SCFA) profile [36]. Firmicutes will perform this rapidly and profligately, as they are adapted to surviving in the acidic conditions they generate, consistent with their food-rich environment. In contrast, members of the Bacteroidetes phylum are less acid tolerant, and will metabolise sugars slower, somewhat maintaining this NAD^+^-requiring redox balance within their cytoplasm, thereby having less impact on their (food-depleted) environment. Their competitive advantage lies with flexibility [37] and priority [38] in terms of choice of substrate. The take-home message from this recap of fundamental metabolism is that the host-derived conditions (dietary carbohydrate, water) drive the microbiome, which in turn drive the conditions (H^+^, pH) in a teleological fashion, in accordance with the scheme outlined in Figure 2. Obviously, secondary metabolites are dependent on this primary metabolism and the microbiome driving it within that faecal snapshot.

How bacteria and their metabolism illustrate the stage and/or location of the gut ecosystem is particularly evident in the case of the H_2_-utilizing bacteria and archaea. When these organisms are the dominant H_2_ utilizer, they tend to maintain H_2_ levels at the lowest threshold they require for growth [39]. Since the methanogen threshold is lower than the acetogen threshold, and the sulphur reducing bacteria threshold is one or more orders of magnitude lower than the methanogen threshold, faecal snapshots really will represent how this cyclic feedback between the factors like transit, water, carbohydrates and the host and microbial influences propagate that local environment at that time and place. Fortunately, this suggests that simply the relative presence or abundance of these H_2_-utilizing microbes and their metabolic activities act as biomarkers that, with sufficient experience, calibration or learning data sets, may allow the investigator to back-calculate or project what the conditions in the rest of the colon are/would be like on the basis of this faecal snapshot.

Examples of successful back-calculation using faecal metabolites is the case of the SCFAs acetate, butyrate and propionate. Butyrate, produced by some Firmicutes, is rapidly absorbed and used by the colonic epithelia as an energy source, which, given the high metabolic activity of the gut, translates to as much as 10% of the body’s energy coming from microbial butyrate. Butyrate is transported into colonocytes and diffuses into the mitochondria where it undergoes β-oxidation to acetyl-CoA which then enters the TCA cycle resulting in reduction of NAD^+^ to NADH, the latter entering the electron transport chain for ATP production [40]. Propionate, predominantly from Bacteroidetes, acts similarly, entering the TCA cycle through succinyl-CoA [40]. The path from ubiquitously produced microbial acetate is directly through acetyl-CoA. This rapid absorption of these acids, particularly butyrate, means that correlations between these and host metabolic markers do not correlate [41]. However, when the uptake or flux of these acids is calculated (parameters obtained through ^13^C acetate, propionate and butyrate infusion of mice caeca), uptake fluxes correlate linearly with host metabolic markers [41]. Known or estimated SCFA flux rates may have potential to assist with correcting or standardizing other metabolite concentrations from faecal snapshots as proxies for colonic contents.

## 3. The Role of Microbiome Structure and Function in Human Health and Disease

Intense clinical investigation of the human gut microbiome has revealed a sophisticated interplay between the microbiome and the host immune system and metabolism. The role of gut microbiome in accomplishing protective, structural and metabolic functions in human hosts is well documented. These include, but are not limited to, defence against colonization by harmful or pathogenic organisms [2,42,43], digestion of food [44,45,46,47], nutrition [46,48,49] and maintenance of a healthy immune system [49,50,51,52,53,54]. Any perturbations in the gut microbiome may result in dysbiosis and can further lead to a variety of phenotypes including obesity, inflammatory bowel disease (IBD), type II diabetes, fatty liver disease, cancer and several additional human disease states or disorders (Table 1). The role of the human gut microbiome in disease development and progression has become a growing research field in the recent years, yet the cause and effect of the gut dysbiosis and human health has not been well documented [2].

The prevalence of technologies (sequencing, databases, and analytical paradigms) that characterise the microbiome community, rather than elucidating mechanisms, has led to an abundance of studies that associate patterns, for instance species diversity or species richness, with disease states. However, despite this focus on community rather than mechanism, robust associations have laid the foundation for further exploration. For example, functional gastrointestinal disorders (FGIDs), defined as disorders of the gut-brain interactions, have been linked, at least partly, to altered gut microbiome and immune dysregulation [71]; however, no clear association between different microbial patterns and FGIDs has been drawn [72,73]. The gut and brain appear to communicate via the neural and hormonal signalling, the immune system, and via microbial metabolites such as SCFAs [74,75], and perturbations of this bi-directional relationship between the colonic microbiome and central nervous system, coined the ‘gut-brain axis’, may manifest in neurological conditions such as anxiety, depression, autism spectrum disorders, and influence mood and social behaviour [74,76,77,78,79,80]. Multifactorial autoimmune diseases have also been explored in the light of the gut microbiome, where the breakdown of intestinal epithelial barrier and failure of the gut mucosal immunity allows for microbial cells or their metabolites to trigger systemic inflammation [81,82,83,84]. The gut microbiome may also activate or inhibit natural, systemic anti-tumour immunosurveillance [85,86] or induce the formation of local cancers [87,88,89], as well as influence the efficacy of some chemotherapy treatments [90,91]. In cardiovascular diseases [92], atherogenic trimethylamine N-oxide (TMAO) produced in the liver from microbially-derived trimethylamine (TMA) and cardioprotective SCFAs [93,94] are of particular interest. An increase in cardiovascular disease, as well as type 2 diabetes risk, has also been linked with metabolic syndrome, which in turn appears to be influenced by the gut microbiome at several levels [95]. The previous notion that a simple change in the ratio of Bacteroidetes to Firmicutes in the human gut contributes to the development of obesity, an aspect of metabolic syndrome, has been recently challenged and appears to be much more complex [96].

## 4. Analysing Microbiome Structure and Function with Non-Metabolomics Approaches

Most of the microbes residing in the gut are strictly anaerobic, so their isolation and cultivation in laboratory conditions is challenging, with almost 75 percent of the gut microbiome uncultivable [97]. Culture independent methodologies such as small subunit (SSU) rRNA gene amplicon sequencing (16S SSU for bacteria, 18S SSU for eukaryotes and ITS in fungi) and whole-metagenome sequencing have helped overcome this limitation to a great extent [98]. The 16S rRNA sequencing is a more rapid method for assessment of overall phylogeny and diversity of a bacterial community. As such, the 16S rRNA method may provide information on the composition of the gut microbiome; however, it does not always provide a clear link between the microbes identified and their functions in the gut [99]. Recent development of next generation sequencing (NGS) tools has greatly advanced the high-throughput metagenomics approach, with several software platforms for comparative analyses on the gene level developed. These include, Integrated Microbial Genomes with Microbiome Samples (IMG/M) [100], MicrobesOnline [101], Microbial Genomes database (MBGD) [102], Roary [103], EzBioCloud [104], OrtholugeDB [105] and Efficient Database framework for comparative Genome Analyses using BLAST score Ratios (EDGAR) [106]. The IMG/M software is one of the largest platforms containing annotated bacterial, archaeal and metagenomic sequence data [107].

Considering the importance of understanding the functional capacity of the microbiome and the low cost-effectiveness of metagenomics approaches, an alternative could be to use the 16S rRNA gene profiles for predicting the functions of the microbial communities [108]. One such predictive tool is Phylogenetic Investigation of Communities by Reconstruction of Unobserved States (PICRUST). This tool is based on over 39,000 reference genomes [109], and relies on the availability of fully characterised bacterial genomes and uses their phylogenetic relationships to predict the functional capacity of other genomes. This tool has been used and validated in the Human Microbiome Project. Odamaki, et al. [110] used PICRUST analysis to study the age-related changes in gut microbiota composition in healthy individuals of various age groups from newborn to centenarian. It is important to understand that while both metagenomics and PICRUST provide a functional hypothesis of the gut microbiome, it still needs validation via the use of specific primers or metabolomic analysis. Tax4Fun is another such tool that predicts functional capabilities for prokaryotes in the Kyoto Encyclopaedia of Genes and Genomes (KEGG) database [111]. The most recent addition to these 16S data analysis pipelines is Piphillin, that overcomes some of the limitations associated with PICRUST, such as its dependence on outdated functional databases and specific data pre-processing tools [99].

A major weakness in 16S research is that 16S profiling is vulnerable to bias from diverse sources. The universal primers are not truly ‘universal’. The universal primer sets tend to underperform when they encounter particular mismatches that undermine hybridization to their target sequence. This may result in the under-amplification of certain organisms. Moreover, they do not capture viruses and archaea [109] or eukaryotes. An overwhelming majority of gut microbiome studies have primarily focused on bacterial flora, to characterize their composition and association with human health and diseases. The gut archeome, mycobiome, virome and eukaryome have received less attention until recently. 

Archaea that reside in the human colon are nearly always strictly anaerobic methanogens; most of which belong to the order *Methanobacteriales* and the most common genera being the closely related *Methanobrevibacter* and *Methanosphaera* [112]. To date, three species of methanogenic archaea have been isolated from human faeces [113]: *Methanobrevibacter smithii* [114], *Methanosphaera stadtmanae* [115] and *Methanomassiliicoccus luminyensis* [116]. *M. smithii* has been found to inhabit in almost 95.7% of humans and is the most abundant methanogen in the human gut. When prevalent, it may control H^2^ concentrations [39]. Bang, et al. [117] recently reported that *M. smithii* and *M. stadtmanae* induce monocyte-derived dendritic cell maturation; *M. stadtmanae* leads to substantial release of pro-inflammatory cytokines in these cells. Lecours, et al. [118] indicated an increased prevalence of *M. stadtmanae* in IBD. Recent studies support an association of *M. smithii* with leanness [119,120,121]. An increased prevalence of methanogens may cause chronic constipation [122]. There is strong evidence that there is a lower prevalence of methanogens in patients that tend to have diarrhoea episodes (such as those with IBD) [123].

The human gut mycobiome is a neglected component of microbiota for several reasons, including lack of stability and low abundance and diversity [3,124]. Interactions between fungi and bacteria are common, but are complex and may have dramatic effects on growth and pathogenesis of micro-organisms [125]. Approximately 247 fungal species belonging to 126 genera have been identified in faeces and GI biopsies [126]. Dollive, et al. [127] found *Aspergillus*, *Cryptococcus*, *Penicillium*, *Pneumocystis* and *Saccharomycetaceae* yeasts (*Candida* and *Saccharomyces*) in the GIT of healthy individuals. Fungi have been associated with a number of GIT diseases including IBD [128,129], peptic ulcers [130], irritable bowel syndrome (IBS) [131], antibiotic-associated diarrhoea [132] and chemotherapy-induced enteric disorders [133].

The enteric virome includes viruses that infect host cells, endogenous retroviruses, and viruses that infect the various microbial inhabitants of the GIT, such as bacteria, archaea, and fungi. As such, there is immense complexity in coding potential of gut virome and has received much less attention as compared to bacterial flora [134]. Bacteriophages are the most abundant and diverse members of gut virome and are most likely to have a substantial impact on the host [135]. The gut virome plays an important role in the pathogenesis of dysbiosis [136]. The gut virome has also been associated with intestinal disorders such as IBD [137], Crohn’s disease (CD) [138] and colon cancer [139].

Many studies investigating the gut microbiome have used metagenomics. Although this is a powerful technology, alone it suffers from the same limitations as other unintegrated omics technologies: (a) inability to identify microbial sources, (b) expensive and time-consuming, (c) presence of human contaminants in samples and (d) lack of functional annotations of outputs [140], although the latter is rapidly improving [141]. With rapid development and integration of the other omics techniques, such as metatranscriptomics, metaproteomics and metabolomics, the functional activity of the gut microbiome can be better identified.

A metatranscriptomics approach is used to study gene activity. Gosalbes, et al. [142] investigated the faecal samples from ten healthy individuals and identified the key functions of gut microbiome—carbohydrate metabolism, energy production and synthesis of cellular components. Several housekeeping functions such as amino acid metabolism and lipid metabolism were under-represented in the gut metatranscriptome. Franzosa, et al. [143] collected the stool samples from eight healthy individuals in order to relate the gut metagenome and metatranscriptome. About 59% of microbial transcripts were differentially regulated relative to their genomic abundances. Sporulation and amino acid biosynthesis were consistently downregulated, and ribosome biogenesis and methanogenesis were consistently upregulated.

Functional activity can be studied using a metaproteomics approach. Verberkmoes, et al. [144] investigated faecal samples from a female healthy monozygotic twin pair by shotgun metaproteomics approach. Several proteins required for translation, energy production and carbohydrate metabolism were identified in faecal samples. Erickson, et al. [145] combined shotgun metagenomics and metaproteomics approaches to identify potential functional signatures of CD in stool samples from six twin pairs that were either healthy, or that had CD. Studies have shown higher similarity in gut microbiota between healthy twins than between unrelated individuals. By contrast, twin pairs in which one or both individuals had CD indicated very dissimilar gut microbiome. Integration of omics approaches revealed ileum CD phenotype was associated with alterations in bacterial carbohydrate metabolism, bacterial–host interactions, as well as human host-secreted enzymes. A study by Kolmeder, et al. [146] revealed that the faecal metaproteome in healthy individuals was subject-specific. The functional metaproteome core was stable over a year and was mainly involved in carbohydrate and degradation.

Table 2 outlines many bioinformatic analytical processes applied to metagenomics and metatranscriptomics data. This information may help us undertake a more holistic approach to understanding the functions of the gut in overall human health, especially in case of diseases such as inflammatory bowel disease, irritable bowel syndrome, and obesity.

## 5. Analysing Microbiome Function with Metabolomics

Metabolomics is ideally placed as the foundation for a systems biology approach in the study of the gut microbiome, primarily because metabolites are involved in biological processes at all levels, driving activity [212] and inter-kingdom communication [213] at the level of the proteome, transcriptome, epigenome, and genome.

Metabolomics provides insights into the molecular mechanisms of microbiome-host intersection which have the potential to be exploited as the predictive tool for dysbiosis, microbiome-metabolome disease signatures, and for the discovery of biomarkers to be used to either diagnose the disease or monitor activity of therapeutics [214]. The symbiotic relationship resulting from the coevolution between microbiota and the human host [215] has been particularly illustrated in the production of SCFA from non-digestible dietary fibres that reach the colon by a range of bacterial species [216]. These volatile metabolites are not only important energy sources for microbial communities and the host, but likely play essential roles in maintaining gut epithelial integrity via tight junction regulation [217]; glucose homeostasis, lipid metabolism and short-term appetite suppression via Peptide YY (PYY) and glucagon-like peptide 1 (GLP-1) signalling pathways [218,219]; and immune function regulation [220,221]. Furthermore, gut microbes are involved in the elimination of toxic compounds [222], synthesis of essential vitamins [223] and may metabolize and influence the bioavailability of other nutritive and non-nutritive components of functional foods and prebiotics [224]. This symbiotic relationship between the gut microbiome, their metabolic products and the human host may be disturbed in disease states [225].

Metabolomics and metabolic profiling are increasingly used in the identification of biomarkers of several GIT disorders including IBD [226,227,228] and colorectal cancer [229]. For instance, increased levels of cadaverine and taurine were found in patients with ulcerative colitis; while higher levels of bile acids and lower concentrations of branched-chain fatty acids were detected in patients with IBS [228]. Finegold, Dowd, Gontcharova, Liu, Henley, Wolcott, Youn, Summanen, Granpeesheh and Dixon [61] identified lower levels of total short-chain fatty acids, including lower levels of acetate, propionate and valerate in children with autism. Some of the main chemical classes that regulate host-gut microbiome interactions are listed in Table 3.

Different analytical strategies have been employed for the quantitative analysis of the metabolome, depending on the availability of the technology and research questions [255]. Metabolites of biological samples, such as serum, plasma, urine, faeces and tissues are different in chemical and physiochemical structure and have a large dynamic range of metabolic concentration. Multiple analytical techniques such as gas chromatography (GC), liquid chromatography (LC) and high/ultra-performance liquid chromatography (H/UPLC) coupled to mass spectrometry (MS), and nuclear magnetic resonance spectroscopy (NMR) enable detection, identification and quantification of a wider range of metabolites [256,257]. There are targeted and untargeted metabolomics approaches, and both have their merits and pitfalls.

A targeted metabolomics approach measures a specific list of metabolites, typically focusing on one or more related pathways of interest [258], driven by a specific biochemical questions or hypothesis. This approach can be effective for pharmacokinetics studies of drug discoveries as well as measuring the influence of the intervention on the targeted pathways or metabolic functions [259]. Targeted metabolomics studies offer distinct advantages for metabolite specificity and quantitative reproducibility.

Untargeted metabolomics, also known as global metabolite profiling, attempts to measure as many metabolites as possible. This approach has enabled new discoveries that link cellular pathways to biological mechanisms and are contributing to the understanding of the cellular metabolism, biology, physiology, medicine and host-microbiome interactions [260,261,262].

In contrast to the targeted metabolomics results, untargeted metabolomics studies generate large amounts of highly complex data. Manual inspection of the thousands of detected picks is impractical and requires instrumental automation with metabolomics software such as MathDAMP, MetAlign, MZMine and XCMS [263,264,265,266]. For the identification of individual metabolites in an untargeted approach, a combination of different techniques is applied to ensure good coverage of the metabolome [256]. Table 4 summarizes the online database available to assist with identification of the individual metabolites generated through NMR and MS detection platforms. These databases contain spectral, chemical, molecular and clinical information about the metabolites found in different human biosamples.

Multidimensional separations based on mass spectrometry are a powerful tool for revealing systems level information [279]. For a multidimensional analysis combining proteomics, metabolomics, lipidomics and glycomics, there are 106 possible proteins, and 200,000 metabolites. If drugs are included, this adds another 1060 compounds, as well as an unknown number of man-made compounds originating from environmental contaminants. Thus, these techniques come with a considerable data burden.

Many microbial metabolites are volatile and their study yields valuable insights into microbial community metabolism, interactions, and inter-kingdom interactions [280,281]. The volatilome is defined as all the volatile compounds that originate from an organism or ecosystem. Studying the blood and faecal volatilome in conjunction with non-volatile metabolites, epigenetic and metagenomic measurements from the same samples can yield valuable insights into metabolism and the interactome. Similarly, the volatile profiles of exhaled breath have been utilized as a technique for phenotyping IBS subjects [282], and breath shows promise as a rapid and non-invasive sample type to rapidly classify phenotype, based on volatile metabolites that may be formed in the gut, transferred to the blood, and then transferred through the lung membrane into the exhaled breath.

## 6. Integrating Multi-Omics Datasets

Integrated multi-omics approaches are challenging because the data obtained from such research consists of two or more matrices that contain the same sample IDs, but a range of different biological variables such as genes, transcripts, metabolites, proteins etc. Based on whether these studies take into consideration of prior knowledge, they can be classified as statistics-based methods or knowledge driven methods. Statistical approaches use univariate or multivariate analysis to understand the correlations between the different datasets. Meanwhile, knowledge-based approaches decipher the potential mechanistic links by using the significant variables identified in the different omics approaches and associating them with an existing knowledge base. They are often presented as interaction networks e.g., metabolic networks [283]. Ultimately, the goal is to triangulate between different biological samples that indicate absorption, secretion, or excretion to decipher the interactive systems at play internally. Chemometrics are important for the integrated analysis of the gut microbiome. Chemometrics is the application of data-driven statistical and computational methods to extract information from the measurement of chemical systems [284]. However, commonly available statistical methods are inadequate when dealing with the multidimensional omics datasets necessary for analysis of the gut microbiome. Thus, new approaches are required to deal with multi-dimensional data [285].

Considering the multi-omics represented by metabolomic and taxonomic profiles require not only linking of the two datasets but should include incorporation of prior reference information about metabolic capacities of community members [286], environmental factors such as nutritional/dietary information, disease, etc. [287]. Integration of data into systems-wide approaches has long been recognized, and in some cases, attempted [97,221,286,287,288,289,290,291,292,293]. However, various challenges with standardization of methodologies include scale [286], chemical complexity [221], financial and human resources [289], ecological and clinical context [294], diet [288], inter-individual variation and noise [291], interactions with other body tissues [290], in vivo access to relevant sampling sites [292], and time scales [295]. Nevertheless, perhaps the most comprehensive attempt to integrate datasets can be illustrated by the Virtual Metabolic Human database (https://www.vmh.life) [287], encompassing tens of thousands of unique reactions, thousands of unique metabolites and human genes, hundreds of thousands of microbial genes, thousands of food types, hundreds of diseases and hundreds of microbes, cross-referenced to more than thirty external database resources, with a high percentage of coverage. This resource and framework may be a first real step in integrating multi-omics information.

Machine learning using algorithms such as support vector machine, random forest, Adaboost, logitboost, neural networks, decision tree and other hybrid methods [296,297] can be applied to these large datasets to aid with interpretation. Although microbiome data are complex, meaningful biological insights have been drawn when applied successfully [298,299,300,301,302]. Saulnier, et al. [303] used supervised learning to classify different subtypes of IBS with a high success rate of about 96%. Hacılar, Nalbantoğlu and Bakir-Güngör [296] used machine-learning analysis to investigate subset of gut microbiota that is associated with IBD. Machine learning with advanced data visualization techniques can reveal patterns not detectable by traditional statistical techniques. It has been applied in diverse places, from the intestinal interactome [304], to prediction of metabolic pathways [305], to integration of metabolomics, lipidomics and clinical data [306]. However, critical to the success of machine learning approaches is the size of the training set. This approach cannot be used on small datasets unless a larger dataset with the same characteristics is available.

NJS16 is an extensive database developed by Sung and co-workers [307]. This literature-curated interspecies network of the human gut microbiota consists of ~570 microbial species and 3 human cell types metabolically interacting via ~44,000 small-molecule transport and macromolecule degradation events. Application of a mathematical model approach to the contents of the global metabolic network was used to extract useful information such as biomarker microbial and metabolic features of the gut microbial ecosystem in type 2 diabetes. This study is a step towards integrative investigations of context-specific community-scale analysis.

Pathway Analysis and Imputation to Relate Unknowns in Profiles from MS-based metabolite data (PAIRUP-MS), recently developed by Hsu et al. [308], is a very exciting approach to analysis of new metabolites and new pathways. PAIRUP-MS enables previously infeasible analyses of a significant portion of signals that often goes unidentified as known metabolites and has been excluded from downstream analyses. This tool, that also offers a pathway annotation and enrichment analysis framework, could possibly be used to link metabolite signals to plausible biological functions despite their unknown chemical identities.

The ability to detect latent variables in omics data, and to separate direct from indirect linkages is key to determining the mechanisms and interactions that drive the gut microbiome [309]. Methods also need to allow environmental and clinical information to be incorporated into the model, to allow multi-factor exploration of interactions within the gut microbiome. Kris Sankaran gives a helpful framework and some insights into future directions for latent variable modelling for the microbiome [310]. Promising approaches include sparsity-based methods (SPLS, Graph-Fused Lasso, CCA), and hierarchical clustering [310]. One such approach currently being applied in a number of studies is Hierarchical-All-against-All analysis [311]. HAllA tests for correlation between all pairs of variables in multi-dimensional datasets and can detect multi-level associations, even in non-homogenous datasets. It has been successfully applied to determine the effect of polyphenolic compounds on the gut microbiome [312].

All of these emerging statistical techniques require considerable computing power compared to traditional statistical analysis, and need to be addressed in new ways. For research institutions in developed countries, this typically means making use of shared research computing infrastructure comprising large computing arrays that can be accessed via virtual machines. Another solution could be carrying out computations via collective, cloud-based computing power. The readers are referred to Huang, Chaudhary and Garmire [301] for a more comprehensive account of various data integration tools.

## 7. Future Directions that Would Accelerate an Integrated Approach

### 7.1. New Sampling Techniques

Under normal gut conditions, the most prevalent gases include methane, carbon dioxide, hydrogen, hydrogen sulphide and nitric oxide. Production of such gases and their relative concentrations affecting gut function may have pathogenic roles in several GIT disorders. Lack of direct access to these gases in the gut limits our understanding of their physiology and functional capacity. Direct sampling of gases inside the GIT is likely to provide much more accurate gas-related biomarkers for physiological abnormalities of the gut. Indigestible, endoscopy capsules have been developed by integrating pH, pressure and temperature sensors (SmartPill^®^, Minneapolis, MN, USA). Kalantar-zadeh, et al. [313] developed indigestible, non-invasive, swallowable intestinal gas capsules that can perform in vivo gas measurements and potentially assess putative gas biomarkers in GIT disorders. The so-called ‘gas capsules’ are obtained by integrating small gas sensors into indigestible capsule platforms. The related clinical procedures are non-invasive and once real-time monitoring of the appropriate locations to be sampled is perfected, these capsules could help in surveying gas-related biomarkers and their concentrations throughout the length of the GIT.

### 7.2. New and Emerging Techniques and Disciplines: Culturomics, the Interactome, Foodomics, and the Exposome

Culturomics is a new approach that uses high-throughput, broad spectrum culturing coupled with mass-spectrometry-based identification [314,315,316]. Culturomics enables exploration of the ‘dark’ microbiome to levels approaching those of pyrosequencing, and is able to identify or characterize microbes present in low concentrations. The use of culturomics in tandem with metagenomics would greatly advance understanding of the gut microbiome.

One new and emerging concept, the interactome, is critical to the advancement of integrated analysis of the gut microbiome. The interactome is defined as the whole set of molecular interactions in a cell [317]. Studying the interactome requires the use of multi-omics data from metabolomics, proteomics, and genomics. However, analysing multi-omics data necessitates the development of new statistical tools, in order to tease out direct from indirect associations. The development and application of such tools to the analysis of the interactome is key to significantly accelerating our understanding of complex interactions in the gut microbiome [225,291,304,307,318,319,320].

The other area critical to the advancement of integrated systems analysis of the gut microbiome is in foodomics. Foodomics is the application of -omics technologies to the study of food and drink, and the nutritional effects of consuming them. It is now well accepted that diet is the main regulator of gut health. Yet, currently, ingested food is not routinely subjected to the same rigorous analytical techniques that faeces are. Dietary recall and food diaries are inadequate in that they are unable to precisely link components from diet with gut health, and important dietary components such as the type of liquids ingested are sometimes ignored. In addition, these techniques do not take into account the degree to which the food has been processed and thus stripped of its natural microbiome and micronutrients, or whether it contains man-made contaminants. Thus, current dietary data collection methods do not accurately reflect the micronutrient or metabolite profile of food as it is prepared and ingested. Metabolite and genomic profiling of food allows investigation of compounds in food and its associated microbiome. While there are few studies that have used a systems-based approach [321], such studies hold great therapeutic potential, as demonstrated by the Ni and co-workers [225] study of interactions between the small molecules from diet and the gut bacterial proteome. Foodomics is an emerging discipline that will become increasingly important to an integrated analysis of the gut microbiome.

Equally critical to the study of the gut microbiome is the integration of the impact of the external environment into systems analysis. The exposome is defined as the totality of environmental exposures from conception onwards. While not all of these factors are easily measured, external contaminants or man-made compounds can be detected using trace analytical techniques, such as mass spectrometry. Analysis of trace environmental contaminants can often be undertaken at the same time as analysis of the metabolome, provided untargeted analytical methods are used, and comprehensive mass spectral libraries such as NIST/Wiley are available to search. At present, primarily due to the availability of large mass spectral libraries, GC-MS is still the most reliable method for exposome analysis. However, considerable advances in analytical software and enlarging databases for high-resolution LC-MS/MS have occurred recently and this technique will surpass GC-MS as many more metabolites are amenable to measurement using LC-MS than GC-MS. Alternatively, targeted analysis of large panels of pharmaceuticals, pesticides, plasticizers, and other man-made compounds can be carried out in tandem with metabolite profiling, but will inevitably suffer from bias due to assumptions made around what compounds to target.

## Figures and Tables

**Table 1:** Changes in the gut microbiome associated with disorder/disease.

Disease/Disorder	Positively Implicated Members of Microbiota (↑)	Negatively Implicated Members of Microbiota (↓)	Reference
Colorectal cancer	*Fusobacterium* *Porphyromonas*	*Clostridium* *Bacteroides* *Lachnospiraceae*	[55]
Colitis-associated colorectal cancer	*Bifidobacterium* *E. coli*		[56]
Cirrhosis	*Enterococcaeae* *Staphylococcaceae* *Enterobacteriaceae*	*Clostridiales XIV* *Ruminococcaceae* *Lachnospiraceae* *Veillonellaceae* *Porphyromonadaceae*	[57]
Non-alcoholic fatty liver disease and steatohepatitis	*Ruminococcus* *Dorea*	*Oscillospira*	[58]
Celiac’s disease	*Bacteroides vulgatus* *Escherichia coli*	*Clostridium coccoides*	[59]
Gastric cancer	*Helicobacter pylori*		[60]
Autism	*Bacteroidetes* *Proteobacteria*	*Actinobacteria* *Firmicutes*	[61,62,63]
Parkinson’s Disease	*Enterobacteriaceae*	*Prevotellaceae*	[64]
Type 2 diabetes	*Betaproteobacteria*	*Firmicutes* *Clostridia*	[65,66]
IBD – Crohn’s Disease	*Bacteroides ovatus* *Bacteroides vulgatus*	*Bacteroides uniformis*	[67]
IBD – Ulcerative colitis	*Gammaproteobacteria* *Deltaproteobacteria* *Actinobacteria* *Proteobacteria*	*Firmicutes*	[68,69,70]

**Table 2:** Computational methods for meta-omic analysis (modified from [98]).

Method	Tool	Description	Reference
*Assembly*	DIME	Combines the DIvide, conquer, and MErge strategies	[147]
	Genovo	Generative probabilistic model of reads	[148]
	Khmer	Probabilistic de Bruijn graphs	[149]
	MAP	OLC (Overlap/Layout/Consensus) strategy for longer reads	[150]
	Meta-IDBA	De Bruijn graph approach	[151]
	metAMOS	A Modular Open-Source Assembler component for metagenomes	[152]
	MetaVelvet	De Bruijn graph approach	[153]
	MOCAT	a metagenomics assembly and gene prediction toolkit	[154]
	SOAPdenovo	Single-genome assembler commonly tuned for metagenomes	[155]
	MetaORFA	Gene-targeted assembly approach	[156]
	MetaPAR	Metagenomic sequence assembly via iterative reclassification	[157]
	XGenovo	An extended Genovo assembler by incorporating paired-end information	[158]
*Taxonomic profiling*	Amphora	Automated pipeline for Phylogenomic Analysis	[159,160,161]
	CARMA3	Taxonomic classification of metagenomic shotgun sequences	[162,163]
	ClaMS	Classifier for Metagenomic Sequences	[164]
	CLARK	Fast and accurate classification of metagenomic and genomic sequences using discriminative k-mers	[165]
	DiScRIBinATE	Distance Score Ratio for Improved Binning and Taxonomic Estimation	[166]
	FOCUS	An agile composition-based approach using non-negative least squares	[167]
	INDUS	Composition-based approach for rapid and accurate taxonomic classification of metagenomic sequences	[168]
	MARTA	Suite of Java-based tools for assigning taxonomic status to DNA sequences	[169]
	MetaCluster	Binning algorithm for high-throughput sequencing reads	[170]
	MetaPhlAn	Profiles the composition of microbial communities from metagenomic shotgun sequencing data	[171,172]
	MetaPhyler	Taxonomic classifier for metagenomic shotgun reads using phylogenetic marker reference genes	[173]
	MOCAT2	A metagenomic assembly, annotation and profiling framework	[174]
	MTR	Taxonomic annotation of short metagenomic reads using clustering at multiple taxonomic ranks	[175]
	NBC	Naive Bayes Classification tool for taxonomic assignment	[176]
	PaPaRa	Aligning short reads to reference alignments and trees	[177]
	PhyloPythia	Accurate phylogenetic classification of variable-length DNA fragments	[164]
	PhyloSift	Phylogenetic analysis of metagenomic samples	[178]
	Phymm	Classification system designed for metagenomics experiments that assigns taxonomic labels to short DNA Reads	[179]
	RAIphy	Phylogenetic classification of metagenomics samples using iterative refinement of relative abundance index Profiles	[180]
	RITA	Classifying short genomic fragments from novel lineages using composition and homology	[181]
	SOrt-ITEMS	Sequence orthology-based approach for improved taxonomic estimation of metagenomic sequences	[182]
	SPHINX	Algorithm for taxonomic binning of metagenomic sequences	[183]
	TACOA	Taxonomic classification of environmental genomic fragments using a kernelized nearest neighbour approach	[184]
	Treephyler	Fast taxonomic profiling of metagenomes	[185]
*Functional profiling*	HUMAnN	Determines the presence/absence and abundance of microbial pathways in meta-omic data	[186]
	metaSHARK	web platform for interactive exploration of metabolic networks	[187]
	MOCAT2	A metagenomic assembly, annotation and profiling framework	[174]
	PRMT	Predicted Relative Metabolomic Turnover: determining metabolic turnover from a coastal marine metagenomic dataset	[188]
	RAMMCAP	Rapid analysis of Multiple Metagenomes with Clustering and Annotation Pipeline	[189]
*Interaction networks*	SparCC	Estimates correlation values from compositional data for network inference	[190]
	CCREPE	Predicts microbial relationships within and between microbial habitats for network inference	[191]
*Single-cell sequencing*	IDBA-UD	De Bruijn graph approach for uneven sequencing depths	[192]
	SmashCell	Software framework for the analysis of single-cell amplified genome sequences	[193]
*Simulators*	GenSIM	Error-model based simulator of next-generation sequencing data	[194]
	Metasim	A sequencing simulator for genomics and metagenomics	[195]
*Statistical tests*	Metastats	Statistical analysis software for comparing metagenomic samples	[196]
	LefSe	Nonparametric test for biomarker discovery in proportional microbial community data	[197]
	ShotgunFunctionalizeR	A statistical test based on a Poisson model for metagenomic functional comparisons	[198]
	SourceTracker	A Bayesian approach to identify and quantify contaminants in a given community	[199]
*General toolkit*	CAMERA	Dashboard for environmental metagenomic and genomic data, metadata, and comparative analysis tools	[200]
	GenBoree	A web-based platform for multi-omic research and data analysis using the latest bioinformatics tools	[201]
	GraPhlAn	Compact graphical representation of phylogenetic data and metadata	[202]
	IMG/M	Integrated metagenome data management and comparative analysis system	[100]
	MEGAN	Software for metagenomic, metatranscriptomic, metaproteomic, and rRNA analysis	[203]
	METAREP	Online storage and analysis environment for meta-omic data	[204]
	MG-RAST	Storage, quality control, annotation and comparison of meta-omic samples	[205]
	Mothur	An open-source software for microbial ecology community analysis	[206]
	QIIME	An open-source bioinformatics pipeline for performing microbiome analysis from raw DNA sequencing data	[207]
	SmashCommunity	Stand-alone annotation and analysis pipeline suitable for meta-omic data	[208]
	STAMP	Comparative meta-omics software package	[209]
	SnoWMan	Pipeline for analysis of microbiome data	[210]
	VAMPS	Visualization and analysis of microbial population structure	[211]

CC BY-NC; Segata et al. Molecular systems biology 2013, 9, 666.

**Table 3:** Metabolites associated with gut microbiome (modified from [230] and [231]).

Metabolite Class	Metabolites	Related Bacteria	Biological Functions	Disease/Disorder	Reference
*Short-chain fatty acids*	acetate, propionate, 2-methylpropionate, butyrate, isobutyrate, hexanoate, valerate, isovalerate	All, although capabilities vary amongst phyla, family and species.	Cholesterol synthesis ↑ (acetate); Gluconeogenesis ↑ (propionate); Energy source for colonocytes ↑ (butyrate); Colonic pH ↓; Growth of pathogens ↓; Water and sodium absorption ↑	Human obesity, insulin resistance and type 2 diabetes, colorectal cancer; cardiovascular disease, IBD - ulcerative colitis, IBD -Crohn’s disease, antibiotic-associated diarrhoea, metabolic syndrome, bowel disorders	[40,232,233]
*Phenolic, Benzoyl and phenyl derivatives*	benzoate, hippurate, phenylacetate, phenylpropionate, 3-hydroxycinnamate, 2-hydroxyhippurate, 3-hydroxyhippurate, 2-hydroxybenzoate, 3-hydroxybenzoate, 4-hydroxybenzoate, 4-hydroxyphenylacetate, 3-hydroxyphenylpropionate, 4-hydroxyphenylpropionate, 3,4-dihydroxyphenylpropionate, 4-methylphenol, 4-cresol, 4-cresyl sulfate, 4-cresyl glucuronide, phenylacetylglycine, phenylacetylglutamine, phenylacetylglycine phenylpropionylglycine, cinnamoylglycine	*Clostridium difficile, Faecalibacterium prausnitzii, Bifidobacterium,* *Subdoligranulum, Lactobacillus*	Detoxification of xenobiotics; indicate gut microbial composition and activity; utilize polyphenols.	Hypertension, Obesity, Colorectal cancer, Autism	[230,234,235,236,237,238,239,240]
*Bile salts*	cholate, hyocholate, deoxycholate, chenodeoxycholate, α-muricholate, β-muricholate, ω-muricholate, taurocholate, glycocholate, taurochenoxycholate, glycochenodeoxycholate, taurocholate, tauro–α–muricholate, tauro–β–muricholate, lithocholate, ursodeoxycholate, hyodeoxycholate, glycodeoxylcholate, taurohyocholate, taurodeoxylcholate	*Lactobacillus, Bifidobacterium,* *Enterobacter, Bacteroides,* *Clostridium,* *Enterobacter, Escherichia*	Absorption of dietary fats and Lipid-soluble vitamins, facilitate lipid assimilation, maintain gut barrier function, regulate triglycerides, cholesterol and glucose by endocrine functions, energy homeostasis.	Colon cancer	[241,242,243]
*Choline metabolites*	methylamine, dimethylamine, trimethylamine, trimethylamine-N-oxide, dimethylglycine, betaine	*Faecalibacterium prausnitzii,* *Bifidobacterium*	Lipid metabolism, Glucose homeostasis	Non-alcoholic fatty liver disease, Dietary-induced obesity, diabetes, cardiovascular disease	[244,245]
*Indole derivatives*	N-acetyltryptophan indoleacetate indoleacetylglycine (IAG) indole indoxyl sulphate indole-3-propionate melatonin melatonin 6-sulfate serotonin 5-hydroxyindole	*Clostridium sporogenes,* *Escherichia coli*	Protect against stress-induced lesions in the GI tract; modulate expression of proinflammatory genes, increase expression of anti-inflammatory genes, strengthen epithelial cell barrier properties	GI pathologies, brain-gut axis, Neurological conditions	[246,247]
*Vitamins*	Vitamin K cobalamin (Vitamin B12) biotin (Vitamin B8) folate (Vitamin B9) thiamine (Vitamin B1) riboflavin (Vitamin B2) pyridoxine (Vitamin B6) niacin (Vitamin B3) pantothenic acid (Vitamin B5)	*Bifidobacterium* Commensal *Lactobacill* *Bacillus* *Subtilis, Escherichia coli* and Anaerobes *Bacteroidetes,* *Fusobacteria* *Proteobacteria* *Actinobacteria*	Cellular metabolism, Provide complementary endogenous sources of vitamins, strengthen immune function, exert epigenetic effects to regulate cell proliferation		[248,249,250,251]
*Polyamines*	putrescine cadaverine spermidine spermine	*Campylobacter jejuni* *Clostridium saccharolyticum*	Exert genotoxic effects on the host, anti-inflammatory In addition, antitumoral effects. potential tumour markers		[252]
*Lipids*	conjugated fatty acids lipopolysaccharide (LPS) peptidoglycan acylglycerols sphingomyelin cholesterol phosphatidylcholines phosphoethanolamines triglycerides	*Bifidobacterium* *Roseburia,* *Lactobacillus* *Klebsiella* *Enterobacter* *Citrobacter* *Clostridium*	Impact intestinal permeability, activate intestine brain- liver neural axis to regulate glucose homeostasis; LPS induces chronic systemic inflammation; conjugated fatty acids improve hyperinsulinemia, enhance the immune system and alter lipoprotein profiles. Cholesterol is the basis for sterol and bile acid production.		[253]
*Others*	D-lactate methanol ethanol formate succinate lysine glucose urea a-ketoisovalerate, creatine creatinine endocannabinoids 2-arachidonoylglycerol (2-AG) N-arachidonoylethanolamide	Bacteroides Pseudobutyrivibrio Ruminococcus Faecalibacterium Subdoligranulum Bifidobacterium Atopobium Firmicutes Lactobacillus	Direct or indirect synthesis or utilization of compounds or modulation of linked pathways including endocannabinoid system.		[237,254]

**Table 4:** Web-based databases for identification of metabolites (modified from [267]).

Database	Web Address/URL	Available Since/Reference
Global Metabolome Database (GMD)	http://gmd.mpimp-golm.mpg.de/	2004, Kopka, et al. [268]
METLIN	https://metlin.scripps.edu/	2005, Smith, et al. [269]
Kyoto Encyclopedia of Genes and Genomes (KEGG)	http://www.genome.jp/kegg/	1995, Kanehisa, et al. [270]
Chemicals Entities of Biological Interest (ChEBI)	http://www.ebi.ac.uk/chebi/	2004, Degtyarenko, et al. [271]
Human Metabolome Database (HMDB)	http://www.hmdb.ca/	2007, Wishart et al. [272,273]
Biological Magnetic Resonance Data Bank (BMRB)	http://www.bmrb.wisc.edu/	2007, Ulrich, et al. [274]
Madison Metabolomics Consortium (MMC) Database	http://mmcd.nmrfam.wisc.edu/	2008, Cui, et al. [275]
BiGG (a knowledgebase of Biochemically, Genetically and Genomically structured genome-scale metabolic network reconstructions)	http://bigg.ucsd.edu/	2010, Schellenberger, et al. [276]
MassBank	http://www.massbank.jp/	2010, Horai, et al. [277]
SetupX and BinBase	https://fiehnlab.ucdavis.edu/	2011, Skogerson, et al. [278]
